# Universal speech foundation model for psychiatric assessment: multi-language validation in mood disorders and psychiatric comorbidities

**DOI:** 10.1192/j.eurpsy.2026.12060

**Published:** 2026-07-31

**Authors:** A. Ando, A. Crabeil, A. Lesage, R. Riad

**Affiliations:** Callyope, Paris, France

## Abstract

**Introduction:**

Speech patterns contain biomarkers reflecting mental health status, offering objective assessment beyond traditional clinical interviews and rating scales. Current speech-based diagnostic tools require extensive patient samples for each psychiatric condition and population, limiting their clinical utility. Developing generalizable speech analysis that works across diverse psychiatric populations without condition-specific training could transform mental health screening and monitoring.

**Objectives:**

To develop and validate SLAP (Speaker contrastive Language-Audio Pretraining), a speech analysis system capable of detecting psychiatric symptoms directly from voice recordings without requiring previous examples of each specific disorder—particularly for depression and suicidality assessment across multiple languages and clinical settings.

**Methods:**

We developed SLAP using machine learning to link speech patterns with clinical descriptions of mental health status. Training utilized 3,415 hours of speech from 13,000+ individuals with psychiatric annotations. We evaluated diagnostic accuracy using balanced sensitivity-specificity measures (combining both false positive and false negative rates for clinical relevance). The system was tested in two modes: immediate assessment without prior disorder-specific training (using clinical descriptors like “depressed” vs. “healthy”), and traditional diagnostic accuracy testing. Evaluation included depression (PHQ-9, MADRS scales), suicidal ideation, anxiety, insomnia, and fatigue across French, Italian, Spanish, and Chinese populations in both community and psychiatric settings.

**Results:**

Without any disorder-specific training, SLAP correctly identified 51% of mental health conditions (balanced accuracy accounting for both missed cases and false alarms), i.e. a 70% improvement over existing automated methods. For depression specifically: 93% accuracy in Italian psychiatric patients (MADRS assessment), 83% in Chinese clinical samples (major depressive disorder), and 70% detecting moderate-severe symptoms (PHQ-9≥10). Crucially, the system maintained accuracy when tested on completely new languages and patient populations never encountered during development.

**Image 1:**
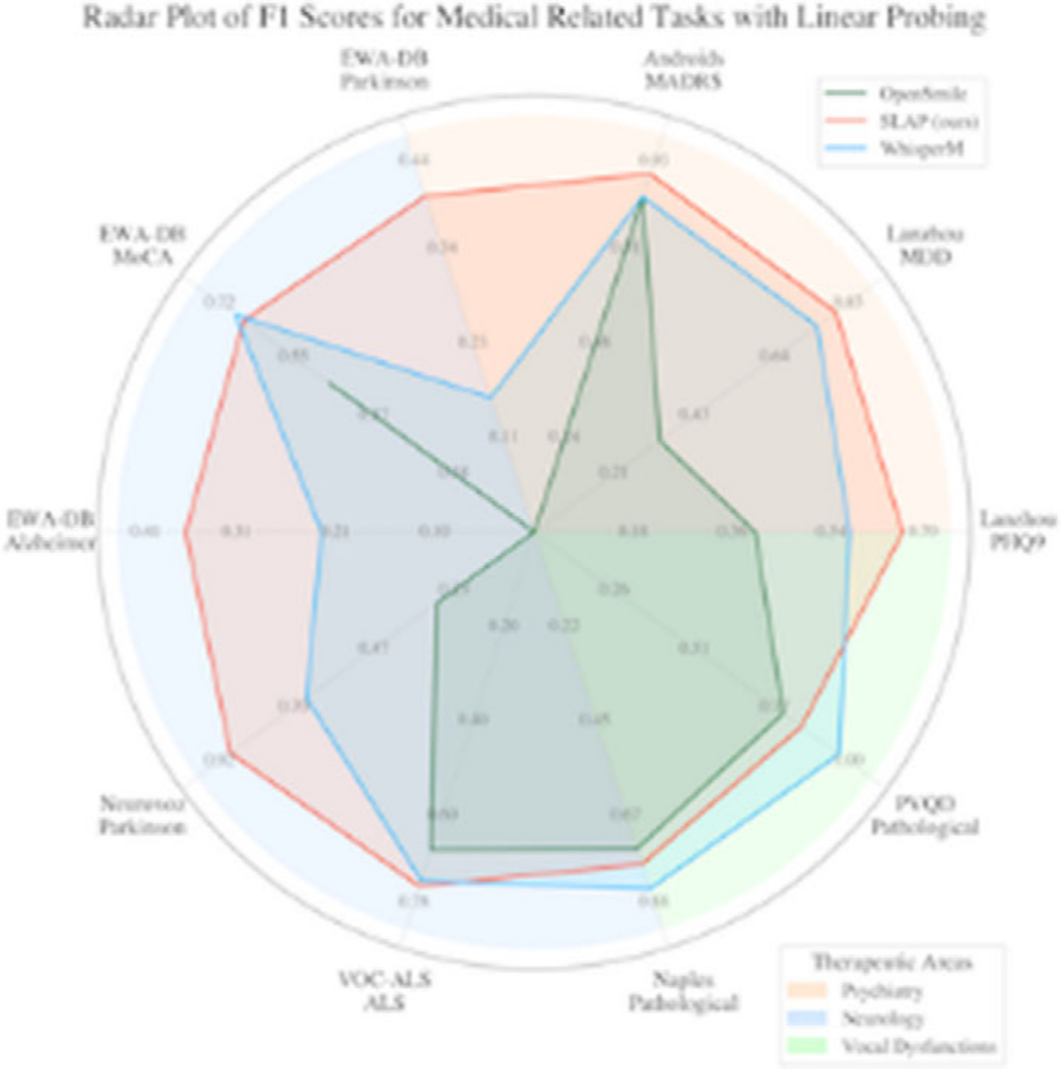
Image 1: Long description.

**Conclusions:**

SLAP enables psychiatric symptom detection from speech without requiring disorder-specific patient databases, addressing a critical barrier to implementing voice-based screening in clinical practice. This technology could enable remote monitoring for relapse prevention, provide objective measures supplementing clinical assessment, and extend mental health screening to underserved populations. The ability to deploy immediately across new languages and populations without collecting new patient data makes this particularly valuable for crisis response and global mental health initiatives.

**Disclosure of Interest:**

A. Ando Shareholder of: Callyope, Employee of: Callyope, A. Crabeil Shareholder of: Callyope, Employee of: Callyope, A. Lesage Shareholder of: Callyope, Employee of: Callyope, R. Riad Shareholder of: Callyope, Employee of: Callyope

## Long descriptions

### Image 1: Long description

A radar chart titled Radar Plot of F 1 Scores for Medical Related Tasks with Linear Probing. The chart features ten axes representing different medical datasets and tasks, categorized into three Therapeutic Areas: Psychiatry (orange background), Neurology (blue background), and Vocal Dysfunctions (green background).

Clockwise from the top, the axes are:

* Androids M A D R S (Psychiatry)

* Lanzhou M D D (Psychiatry)

* Lanzhou P H Q 9 (Psychiatry)

* P V Q D Pathological (Vocal Dysfunctions)

* Naples Pathological (Vocal Dysfunctions)

* V O C dash A L S A L S (Neurology)

* Neurovoz Parkinson (Neurology)

* E W A dash D B Alzheimer (Neurology)

* E W A dash D B M o C A (Neurology)

* E W A dash D B Parkinson (Neurology)

Three data series are plotted:

1. S L A P (red line): Shows the highest overall performance, maintaining a broad, nearly circular shape near the outer edges of the chart, with peaks around 0.90 for Androids M A D R S and 0.78 for V O C dash A L S.

2. Whisper M (blue line): Shows moderate performance, generally following the shape of S L A P but retracted toward the center, with a notable dip in the Psychiatry sector.

3. OpenSmile (green line): Shows the lowest performance, with a highly irregular shape concentrated mostly in the Vocal Dysfunctions and Neurology sectors, and very low scores (near 0.10 to 0.20) in the Psychiatry sector.

Navigate back to Image 1:.

